# Genes of Different Catabolic Pathways Are Coordinately Regulated by Dal81 in *Saccharomyces cerevisiae*

**DOI:** 10.1155/2015/484702

**Published:** 2015-09-17

**Authors:** Marcos D. Palavecino, Susana R. Correa-García, Mariana Bermúdez-Moretti

**Affiliations:** Departamento de Química Biológica, Facultad de Ciencias Exactas y Naturales, Universidad de Buenos Aires, IQUIBICEN-CONICET, Ciudad Universitaria, 1428 Buenos Aires, Argentina

## Abstract

Yeast can use a wide variety of nitrogen compounds. However, the ability to synthesize enzymes and permeases for catabolism of poor nitrogen sources is limited in the presence of a rich one. This general mechanism of transcriptional control is called nitrogen catabolite repression. Poor nitrogen sources, such as leucine, *γ*-aminobutyric acid (GABA), and allantoin, enable growth after the synthesis of pathway-specific catabolic enzymes and permeases. This synthesis occurs only under conditions of nitrogen limitation and in the presence of a pathway-specific signal. In this work we studied the temporal order in the induction of *AGP1, BAP2, UGA4*, and *DAL7*, genes that are involved in the catabolism and use of leucine, GABA, and allantoin, three poor nitrogen sources. We found that when these amino acids are available, cells will express *AGP1* and *BAP2* in the first place, then *DAL7*, and at last *UGA4*. Dal81, a general positive regulator of genes involved in nitrogen utilization related to the metabolisms of GABA, leucine, and allantoin, plays a central role in this coordinated regulation.

## 1. Introduction

Yeast cells can use a variety of compounds as nitrogen source through different specific pathways. Synthesis of proteins involved in each of these pathways is tightly regulated. The availability of readily transported and metabolized nitrogen sources, which are known as good nitrogen sources, results in the strong repression of genes involved in transport and metabolism of poor ones [1, 2]. The utilization of poor sources such as leucine and other branched amino acids, *γ*-aminobutyric acid (GABA), and allantoin requires the synthesis of pathway-specific catabolic enzymes and permeases. This synthesis occurs only under conditions of nitrogen limitation and in the presence of a pathway-specific signal such as a substrate or an intermediate of a metabolic pathway [3].

Extracellular GABA is imported into* Saccharomyces cerevisiae* cells through three proteins: the general amino acid permease (Gap1), the proline specific permease (Put4), and the GABA permease (Uga4) [4]. Within the cells, GABA is catabolized by the GABA transaminase and succinate semialdehyde dehydrogenase enzymes encoded by* UGA1* and* UGA2* genes, respectively [5]. The induction of* UGA* genes by GABA requires at least two positive regulatory proteins: the specific Uga3 factor and the pleiotropic Dal81 factor (also called Uga35) that act through a 19 bp CG-rich Upstream Activating Sequence named UAS_GABA_ [6–8]. The UAS_GABA_ present in* UGA4* and* UGA1* promoters contains two independent Uga3 binding sites that consist of the asymmetric sequence 5′-SGCGGNWTT-3′ (S = G or C, W = A or T, and N = no nucleotide or G) whereas* UGA2* promoter contains only one site [9, 10]. Both factors Uga3 and Dal81 interact* in vivo* with the* UGA4* promoter in a GABA-dependent manner [11].

Leucine is a major amino acid in nutrients and proteins. Its uptake is mediated by multiple amino acid permeases, including the high-affinity leucine permeases Bap2 and Bap3 and the high capacity permease Agp1. When leucine is available in the environment, the SPS (Ssy1, Ptr3, and Ssy5) sensor is activated and hence the transcriptional induction mediated by the homologous transcription factors Stp1 and Stp2 takes place. These factors bind to the sequence known as* UASaa* first identified in the* BAP3* promoter [13] and then in the* BAP2* promoter [14] but later also found in the promoter sequences of several amino acid permease genes including* AGP1 *[15]. In the absence of amino acids, Stp1 and Stp2 are present mainly in the cytoplasm. When amino acids are detected in the environment, 10 kDa of the N terminus of these transcription factors is endoproteolytically cleaved off, resulting in their relocation to the nucleus [16]. Then, Stp1 and Stp2 can act on* AGP1* and* BAP2* promoters. Dal81 acts through the* UAS* sequence facilitating the binding of Stp1 and Stp2 to* AGP1* promoter [17, 18]. It was proposed that Dal81 is also essential for the induction of* BAP2* [19] and that Leu3 modifies the expression of* BAP2* [14].

Allantoin, the heterocyclic compound produced during purine degradation, is rich in nitrogen, and* S. cerevisiae* among many other organisms is able to degrade it and recycle it in order to use it as nitrogen source. The allantoin degradation pathway converts allantoin to ammonia and carbon dioxide. Conversion of allantoin to ammonia is carried out by the* DAL1*,* DAL2*, and* DAL3* gene products, which work sequentially to generate urea. Urea is then degraded to ammonia in a two-step process by the Dur1,2 protein, a multifunctional single enzyme originally thought to be encoded by two tightly linked genes.* DAL7* encodes for an enzyme recycling the glyoxylate generated during allantoin degradation. All the enzymes associated with allantoin degradation are inducible and repressible. Their production is contingent on the presence of allophanate, the last intermediate in the allantoin-degradative pathway or the gratuitous inducer, oxaluric acid [20]. The cis-acting element mediating inducer responsiveness of the allantoin pathway genes is the dodecanucleotide element *UIS*_ALL_(Upstream Induction Sequence). The* DAL81* and* DAL82/DURM* gene products are required for this inducer responsiveness. Dal82 has been shown to be the *UIS*_ALL_ DNA-binding protein whose binding to DNA is independent of inducer. Whereas Dal82 appears to be a pathway-specific regulatory element, Dal81 functions more broadly, being required for induced expression of the* UGA* (GABA catabolic pathway) and* AGP1* (amino acid uptake) genes as well as those of the allantoin pathway [21].

Dal81 is a general positive regulator of genes involved in nitrogen utilization related to metabolisms of GABA, urea, leucine, and allantoin [22, 23]; moreover, Dal81 is involved in the amino acid SPS sensor pathway [17, 18, 24]. In all these induction processes, Dal81 acts together with an inducer-specific protein; this specific factor is Uga3 in GABA-induction of* UGA *genes [6, 11, 25], Dal82 in allophanate-induction of* DUR* and* DAL* genes [26–28], and Stp1 in amino acid induction of amino acid permease genes such as* BAP2*,* BAP3*, and* AGP1* [17, 18, 24].

A hierarchy for different inducible processes mediated by the Dal81 factor has been proposed [24]. Our results showing that the decrease caused by leucine in the recruitment of HA-Dal81 to the* UGA4 *promoter depended on Ssy1 supported this hypothesis [11]. The aim of this work was to study the mechanisms that lead to the temporal order in the expression of genes involved in the use of poor nitrogen sources.

## 2. Methods

### 2.1. Strains and Media

The* S. cerevisiae* strains used in this study are isogenic to the wild type Σ1278b and are listed in Table 1.

Cells were grown in minimal medium containing 0.17% Difco yeast nitrogen base (YNB without amino acids and ammonium sulfate) with 2% glucose as carbon source and 10 mM proline as nitrogen source. The final concentrations of the inducers GABA, leucine, and oxalurate were 0.1 mM, 1.3 mM, and 1 mM, respectively.

MPY09, DEBY01, and DEBY02 mutant strains were generated using the PCR-based gene-deletion strategy [29, 30] or modified versions of it [31].

The MPY09 strain (*leu3*Δ deletion) was generated using the pUG6 plasmid [32] to amplify the* loxP-KanMX-loxP* cassette with primers F-*leu3* and R-*leu3* (Table 2). After generating the strain,* KanMX* cassette was excised by recombination mediated by Cre recombinase (pSH47 plasmid).

DEBY01 and DEBY02 strains were generated using pFA6a-3HA-KanMX6 plasmid [31] and the F-*STP1-HA* and R-*STP1-HA* primers (Table 2). The correct insertion of the tag was corroborated by PCR using the F-*STP1 *int, R-*STP1 down, *R-Kan int primers.

All yeast transformations were carried out using the lithium method [33]. Transformants were selected on rich medium containing 200 *µ*g/ml G418.

To complement the deficiency in* DAL81* gene, cells were transformed with the pSBC-HA-DAL81 plasmid that contains the complete* DAL81* gene [34].

### 2.2. Quantitative RT-PCR

RT-qPCR experiments were performed according to Cardillo et al. [34]. cDNAs were quantified by RT-PCR using an Opticon Monitor 3 (Bio-Rad) with the primers listed in Table 2. Expression values correspond to the ratio of concentrations of* UGA4*,* AGP1*,* BAP2*, and* DAL7* over* TBP1* specific mRNAs determined in each sample and represent the mean ± SEM of three independent experiments.

### 2.3. Chromatin Immunoprecipitation Assays

Chromatin immunoprecipitation (ChIP) experiments were performed according to Cardillo et al. [25]. Normal mouse IgG (Santa Cruz) or monoclonal anti-HA antibody (HA probe (F-7), Santa Cruz) was used. Real time quantitative PCR was carried out in an Opticon Monitor 3 (Bio-Rad) with primers that amplified promoter regions of* UGA4, AGP1, DAL7, *and* BAP2* genes (Table 2). A pair of primers that amplified a region located 2.5 Kb downstream of* UGA4* promoter (F-UC/R-UC) was used as an unbound control. ChIP DNA was normalized to input DNA and calculated as a signal-to-noise ratio over IgG control ChIP. The ΔΔCt method was used to calculate fold change of binding to the promoter of interest [35]. Results are expressed as the mean ± SEM of three independent experiments.

## 3. Results and Discussion

It has been proposed that the expression of genes that encode permeases of different poor nitrogen sources follows a certain order rather than occurring simultaneously.

**Figure 1:**
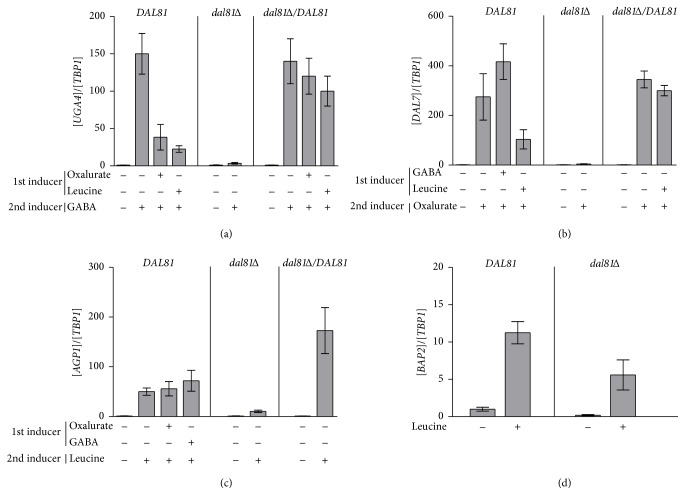
Effect of Dal81 on gene induction. mRNA levels of* UGA4 *(a),* DAL7* (b),* AGP1* (c), and* BAP2* (d) were determined in wild type cells (23344c strain),* dal81*Δ cells (SBCY17 strain), and* dal81*Δ cells transformed with the pSBC-HA-DAL81 plasmid. The second inducer, the specific one, was added 30 minutes after the addition of the first one and cells were incubated for another 30 minutes. mRNA levels were quantified by RT-qPCR.* UGA4*,* DAL7, AGP1,* and* BAP2* values were normalized with* TBP1* and results are the mean ± SEM of three independent experiments. Within each strain the values measured were normalized to the value obtained for the uninduced condition.

In order to determine this hierarchy, we measured the induction of each gene in the presence of the inducer of another gene. For instance, to analyze the effect of the inducers of* DAL7*,* AGP1,* and* BAP2* genes on GABA-induction of* UGA4*, exponentially grown cells were incubated for 30 minutes with oxalurate or leucine before the 30-minute incubation with GABA. The induction of* UGA4* produced by the presence of GABA was strongly inhibited by leucine, the inducer of* AGP1* and* BAP2* genes, and also by oxalurate, the inducer of* DAL7 *(Figure 1(a)). This is indicating that GABA is incorporated into the cells for its catabolism only after the leucine and allantoin added to the culture medium are used. The induction of* DAL7* by oxalurate was significantly lower when cells were previously incubated by leucine while preincubation with GABA did not produce any effect (Figure 1(b)), suggesting that the use of allantoin might occur in the presence of GABA but only after the consumption of leucine. The expression of* AGP1* and* BAP2* genes was induced by leucine and it was not affected by GABA or oxalurate (Figures 1(c) and 1(d)). These results suggest that the order of the use of the analyzed poor nitrogen sources could be leucine, allantoin, and then GABA.

As expected, the induction of* UGA4*,* DAL7,* and* AGP1 *by GABA, oxalurate, and leucine, respectively, strictly depended on the activity of the transcription factor Dal81 since no induction was detected in* dal81*Δ cells (Figures 1(a), 1(b), and 1(c)). On the other hand, the induction of* BAP2* measured in* dal81*Δ cells was significantly lower than that observed in wild type cells although some induction was still detected (Figure 1(d)). Our results suggest that although Dal81 is involved in* BAP2* transcription as it was reported earlier [19], it is not essential for this process. Both* BAP2* and* AGP1* are regulated by the pair of transcription factors Dal81 and Stp1; however the role of Dal81 on the regulation of each gene seems to be different.

The fact that Dal81 is involved in these induction processes makes this factor a good candidate as the protein that establishes the hierarchy. So, this hierarchy might be due to the recruitment of Dal81 in the firstly induced genes with the consequently lower availability of this transcription factor to act on the other promoters. To test this hypothesis, we measured the induction of each gene in the presence of the inducers of other genes in cells that overexpressed Dal81. For this purpose,* dal81*Δ cells were transformed with the pSBC-HA-DAL81 plasmid [34] that contains the complete* DAL81* open reading frame under the control of the* GPD1 *promoter. When the expression of* DAL81* was under the regulation of the strong and constitutive promoter* GPD1*, the induction of* UGA4* by GABA was not inhibited by the presence of leucine nor oxalurate and the induction of* DAL7* by oxalurate was not inhibited by the presence of leucine (Figures 1(a) and 1(b)). So, this finding confirms our hypothesis since when Dal81 is not a limiting factor, the hierarchy is not observed.

**Figure 2:**
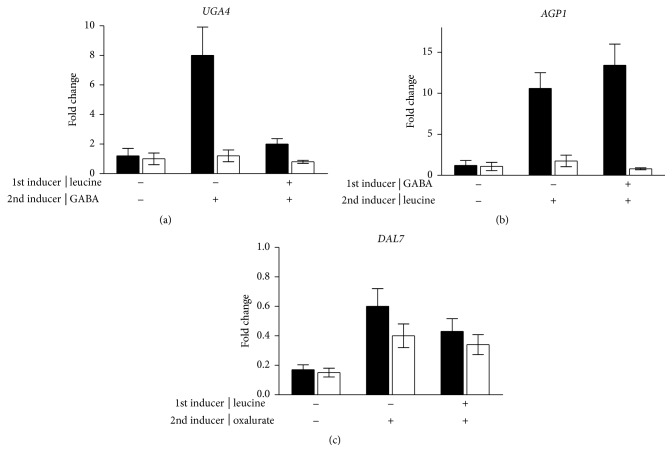
Interaction of Dal81 with* UGA4, AGP1,* and* DAL7 *promoters. Wild type cells, expressing the HA-Dal81 (SBCY10 strain) fusion protein, were treated or not with the indicated inducers. ChIP assays were carried out using antibodies against the HA epitope. qPCR was performed with specific primers (black bars) that amplify a region of* UGA4* promoter (a), a region of* AGP1* promoter (b), a region of* DAL7* promoter (c), and a region 2.5 kb downstream of* UGA4* promoter used as a negative control (white bars). Results are expressed as the fold change of binding to the promoter of interest and are the mean ± SEM of three independent experiments.

Then we analyzed the binding that occurs* in vivo* between Dal81 and* UGA4, AGP1, *and* DAL7* promoters. We found that the strong binding of Dal81 with the regulatory region of* UGA4* detected after incubating the cells with GABA significantly weakened when the cells were preincubated with leucine (Figure 2(a)). This inhibition by leucine was earlier shown [11]. In the contrary, the amount of Dal81 bound to the promoter of* AGP1* did not change after the preincubation with GABA (Figure 2(b)). These results reinforce our idea of Dal81 being the limiting factor in these induction processes. On the other hand, we did not find any effect of leucine on the oxalurate-dependent binding of Dal81 to* DAL7* promoter (Figure 2(c)). However, it must be noticed that the binding measured after the incubation with oxalurate was already very weak. So, we think that this result is probably due to technical difficulties in these ChIP assays.

**Figure 3:**
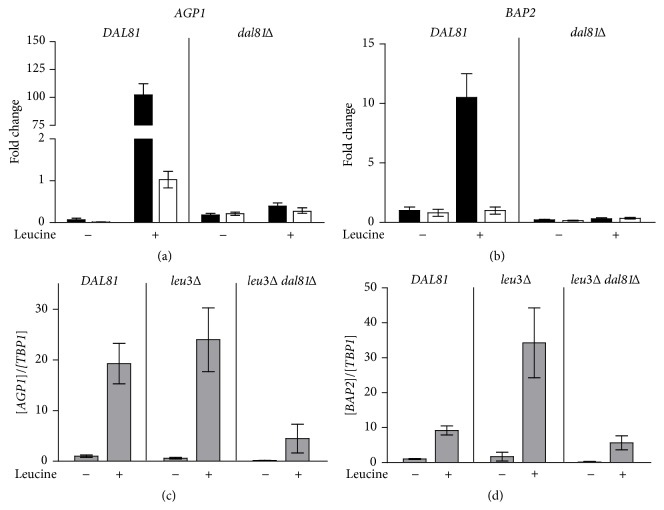
Role of Dal81 in* AGP1* and* BAP2* regulation. Wild type cells, expressing the Stp1-HA (DEBY01 strain) fusion protein, were treated or not with leucine. ChIP assays were carried out using antibodies against the HA epitope. qPCR was performed with specific primers (black bars) that amplify a region of* AGP1* promoter (a), a region of* BAP2* promoter (b), and a region 2.5 kb downstream of* UGA4* promoter (white bars) used as a negative control. Results are expressed as the fold change of binding to the promoter of interest and are the mean ± SEM of three independent experiments. mRNA levels of* AGP1* (c) and* BAP2* (d) were determined in wild type cells (23344c strain),* leu3*Δ cells (MPY09 strain), and* leu3*Δ* dal81*Δ cells (SBCY20 strain). The cells were incubated with or without leucine for 30 minutes. mRNA levels were quantified by RT-qPCR.* AGP1* and* BAP2* values were normalized with* TBP1* and results are the mean ± SEM of three independent experiments.

We were not able to detect Dal81 bound to the regulatory region of* BAP2* gene (data not shown). This suggests that the mild effect of Dal81 detected on* BAP2* (Figure 1(d)) is not due to a direct interaction. Again, Dal81 acts in a different way on* BAP2* compared to on* AGP1* in contrast with previous reports [18]. To assess this, we measured the binding of Stp1 to* AGP1* and* BAP2* genes. We found that Stp1 strongly bound to* AGP1* promoter after the incubation with the inducer leucine and this interaction was avoided in a* dal81Δ* strain (Figure 3(a)). This result is in agreement with those obtained by Boban and Ljungdahl [17]. Stp1 also bound to* BAP2* promoter in a leucine dependent manner (Figure 3(b)). However, it was rather surprising to find that this interaction also depended on the presence of Dal81. As Dal81 did not seem to bind to* BAP2*, the mechanism by which it facilitates Stp1 binding to this gene might be indirect, probably through Leu3. The expression of* BAP2* gene was significantly higher in* leu3*Δ cells, indicating that in our growth conditions Leu3 factor has a negative effect on* BAP2* (Figure 3(d)). It was earlier reported that the Leu3 binding site in the* BAP2* promoter is required for full promoter activity [14]. This apparent contradiction is probably due to different growth conditions since it is known that Leu3 is a transcription factor that can act as an activator or as a repressor depending on the content of *α*-isopropylmalate within the cells [36]. Different growth conditions lead to changes in this content. Our results also reveal a substantial difference in the regulation of* AGP1* and* BAP2* since* AGP1* is not regulated by Leu3. In the* leu3*Δ* dal81*Δ double mutant the expression of* BAP2* was significantly lower than in the single mutant* leu3*Δ, confirming that Dal81 is somehow participating in the expression of* BAP2*. It must be noted that we have already found a negative effect of Leu3 on* UGA4 *gene, although we were not able to detect Leu3 bound to this gene [11]. Moreover, De Boer et al. demonstrated that Leu3 acts as a repressor on* BAP3* expression but they also failed in detecting any interaction [13].

## 4. Conclusions

In this work we showed that the expression of the proteins responsible for the uptake and catabolism of different poor nitrogen sources occurs sequentially following a certain order determined by a tight regulation. This order could promote the utilization of a given nitrogen source whereas the utilization of others, which may be less useful, could be downregulated in some growth media. We demonstrated here that the transcription factor Dal81 is central in the regulation that leads to the hierarchical expression of the genes studied here and, consequently, in the utilization of leucine, allantoin, and GABA.* BAP2*, the gene of one of the permeases of leucine, seems not to be included in this hierarchical regulation since the regulation of* BAP2 *by Dal81 occurs through an indirect mechanism. This may have a physiological significance since Iraqui and collaborators [18] failed to show any contribution of Bap2 to the utilization of leucine as the sole nitrogen source and Cohen and Engelberg [37] suggested that Bap2 is the only functional leucine transporter on rich media.

The doubling time of wild type cells in minimal medium-cultures containing leucine as the sole nitrogen source was twice that of cultures in minimal medium containing GABA (http://www.doubling-time.com/compute.php). Other authors obtained similar values of doubling time of cells grown on allantoin or GABA as the sole nitrogen sources [38]. It is commonly assumed that preferred nitrogen sources allow higher growth rates. This is true when comparing rich and poor sources. However, if the order of the expression of* AGP1*,* DAL7,* and* UGA4* was determining a hierarchy in the use of their substrates, that assumption would not be fulfilled for poor nitrogen sources. Probably, the importance of leucine as a ketogenic amino acid that supplies energy to the cells and its high frequency in protein composition led to a regulation that ends in the use of this poor nitrogen source before others that provide a higher growth rate.

## Figures and Tables

**Table 1:** Strains used in this work.

Strain	Genotype	Parent	Primer	Source or reference
Σ1278b	*Matα*	—	—	[12]
23344c	*Matα ura3*	—	—	Grenson et al. [4]
SBCY10	*Matα ura3 6HA-DAL81 *	—	—	[11]
SBCY17	*Matα ura3 dal81*Δ*::natMX4*	—	—	[11]
SBCY20	*Matαura3 uga35*::*natMX4 leu3*::*KanMX4*	—		[11]
MPY09	*Matα ura3 leu3::loxP*	23344c		This study
DEBY01	*Matα ura3 STP1-3HA-KanMX6*	23344c		This study
DEBY02	*Matα ura3 dal81*Δ*::natMX4 STP1-3HA-KanMX6*	SBCY17		This study

**Table 2:** Primers used in this work.

Primer group and name	Sequence (5′ to 3′)
Oligonucleotides for strain construction	
F-*leu3*	TGCAATTATGGAAGGAAGATCAGATTTTGTGGCGACTTCACACAGCTGAAGCTTCGTACGC
R-*leu3*	GGACTTTAAACCTTGGGATTGAACGCAAATTCATTCATTAAACATAGGCCACTAAGTGGATCTG
F-*STP1*-HA	CAATATTTGAATTTTTACAATGACAACTTTGGGTCACAATTTCGGATCCCCGGGTTAATTAA
R-*STP1*-HA	TTCCAATATGATACCCTTATTTTTATCCCGTGTTATATTTAAGAATTCGAGCTCGTTTAAAC
F-*LEU3* prom	AGGTGCCGCCTAATTTATCG
R-*LEU3* int	ACTTCTGCTGACGACATTCC
F-KanMX6 int	CATCCTATGGAACTGCCTCG
R-KanMX6 int	GATAGATTGTCGCACCTGATTG
F-*STP1 *int	GCACAAGATAATCCTTCGTTCC
R-*STP1 down*	TCGGCTTTCCAATATGATACCC
R-Kan int	CTATACCTGAGAAAGCAACCTG

Oligonucleotides for RT-qPCR	
F-qRT-*UGA4*	CTGCTGCTGTCACATTAACC
R-qRT-*UGA4*	AATACACATAACCACCACTGC
F-qRT-*DAL7*	AACCGAACAAATCAGGAAC
R-qRT-*DAL7*	CAAGTTGGAGATGAAGAGTC
F-qRT-*BAP2*	TAGAGGATGGCGTTGAGTC
R-qRT-*BAP2*	ACCAAGATGTAACCAATTATTAGC
F-qRT-*AGP1*	ATCTTATTCCTATTCTTGGCTACC
R-qRT-*AGP1*	CGGCGTTAATGAAGTGTGG
*F-qRT-TBP1*	TATAACCCCAAGCGTTTTGC
*R-qRT-TBP1*	GCCAGCTTTGAGTCATCCTC

Oligonucleotides for ChIP	
F-*UGA4*	GGAACTGATTACTGTGCCAAG
R-*UGA4*	AATCGCTTATCGCTTATCGTG
F-*AGP1*	TTATACCTCGGCGGCTTC
R-*AGP1*	GCAAGATTTCTCCAAAGTCC
F*-BAP2*	AGGAGGCTACTGACACTGC
R-*BAP2*	GCTGACATATTTACCGTTGAAGG
F-*DAL7*	AATCTCCGCTGAAGTTGC
R-*DAL7*	TTTCACGATGTACCTTATCCAAGA
F-*UC*	AGTCCAATACCTCTGTCCTC
R-*UC*	AGCCGCAACTTCATTCTG
